# Nuclear Magnetic Resonance seq (NMRseq): A New Approach to Peptide Sequence Tags

**DOI:** 10.3390/toxins10110437

**Published:** 2018-10-28

**Authors:** David Wilson, Norelle L. Daly

**Affiliations:** Centre for Biodiscovery and Molecular Development of Therapeutics, AITHM, James Cook University, Cairns, QLD 4870, Australia; david.wilson4@jcu.edu.au

**Keywords:** NMR spectroscopy, disulfide-rich peptide, conotoxin

## Abstract

Structural analysis of peptides with nuclear magnetic resonance (NMR) spectroscopy generally relies on knowledge of the primary sequence to enable assignment of the resonances prior to determination of the three-dimensional structure. Resonance assignment without knowledge of the sequence is complicated by redundancy in amino acid type, making complete de novo sequencing using NMR spectroscopy unlikely to be feasible. Despite this redundancy, we show here that NMR spectroscopy can be used to identify short sequence tags that can be used to elucidate full-length peptide sequences via database searching. In the current study, we have used this approach to identify conotoxins from the venom of the cone snail *Conus geographus* and determined the three-dimensional structure of a member of the I3 superfamily. This approach is most likely to be useful for the characterization of disulfide-rich peptides, such as those that were chosen for this study, as they generally have well-defined structures, which enhances the quality of the NMR spectra. In contrast to other sequencing methods, the lack of sample manipulation, such as protease digestion, allows for subsequent bioassays to be carried out using the native sample used for sequence identification.

## 1. Introduction

Peptides are highly prevalent in nature and they are of particular interest as therapeutic and bioinsecticide leads due to their typically improved stability, ease of production, and reduced antigenicity over proteins [1,2,3,4]. Characterization of the structures and functions of peptides is required to assess the therapeutic or insecticidal potential of a peptide, but before these studies can be carried out it is essential to determine the amino acid sequence. Historically, the sequence has been obtained through chemical sequencing technologies, such as Edman degradation. More recently, advances in omics technologies, such as genomics, transcriptomics, and proteomics have allowed for relatively rapid elucidation of vast numbers of peptide sequences through an integrated bioinformatic approach [5]. However, none of these technologies allow for non-destructive determination of the sequence of the peptide in its native form.

Here, we introduce NMRseq, a complementary proteomics workflow, which allows for the non-destructive determination of sequence tags of a peptide in the native form, while simultaneously providing data on the three-dimensional structure of the molecule. This approach involves the analysis of standard two-dimensional nuclear magnetic resonance (NMR) spectra, including COSY, TOCSY, HSQC, and NOESY, and it is highly suited to the analysis of small peptides with well-resolved cross-peaks in the NMR spectra, such as venom peptides. Venoms from organisms, such as cone snails and spiders, are rich sources of disulfide-rich peptides, many of which have well-defined structures and are selective and potent modulators of ion channels or receptors [6,7]. In the current study, we have exemplified the NMRseq approach through the analysis of two peptides from the cone snail venom from the venomous marine mollusc *Conus geographus*. Furthermore, we have determined the three-dimensional structure of a member of the I3 superfamily of conotoxins.

## 2. Results

### 2.1. Venom Isolation and Purification

**Figure 1:**
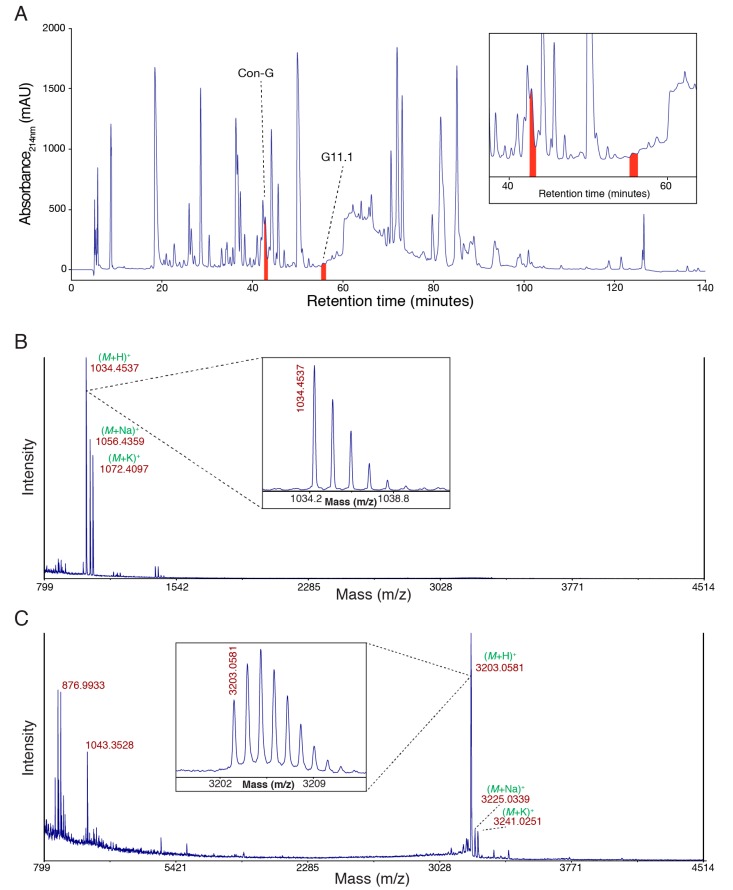
Characterisation of *Conus geographus* venom peptides. (**A**) Semi-preparative reversed-phase high performance liquid chromatography (RP-HPLC) chromatogram of crude *Conus geographus* venom with the peaks corresponding to the two peptides conopressin G (Con-G; mass 1033.4459 Da) and G11.1 (mass 3202.0503 Da), respectively, highlighted in red (C_18_ Phenomenex Jupiter 250 × 10 mm, 10 µm, 300 Å; 3 mL/min flow rate; 0–60% Solvent B in 120 min (Solvent A: 0.05% trifluoroacetic acid (TFA); Solvent B: 90% acetonitrile, 0.045% TFA), 60–90% Solvent B in 5 min, 90% Solvent B for 10 min, 90–0% Solvent B in 5 min; UV absorbance at 214 nm and 280 nm); (**B**) SCIEX TOF/TOF™ 5800 matrix-assisted laser desorption ionization (MALDI) MS spectrum of the conopressin G fraction (mass 1033.4459 Da) using α–cyano-4-hydroxycinnamic acid (CHCA) matrix with the isotope distribution shown expanded inset; (**C**) SCIEX TOF/TOF™ 5800 MALDI MS spectrum of the G11.1 fraction (mass 3202.0503 Da) using CHCA matrix with the isotope distribution shown expanded inset.

Defense-evoked venom was milked from *C. geographus* and fractionated using semi-preparative reversed-phase high performance liquid chromatography (RP-HPLC) (Figure 1A). The profile is complex, as consistent with previous analyses of venom from this species [8]. Two peaks, each corresponding to the purified peptides characterised in this study, are highlighted. The masses of these two peptides correspond to 1033.4459 Da and 3202.0503 Da, respectively. These peptides were chosen because they were collected in relatively homogeneous fractions from the RP-HPLC and they were purified in sufficient quantities for subsequent analyses.

### 2.2. Elucidation of the NMR Sequence Tag for Conopressin G

The peptide corresponding to a mass of 1033.4459 Da was dissolved in aqueous solution at a concentration of approximately 0.5 mM and two-dimensional NMR spectra, including TOCSY, NOESY, COSY, and ^13^C HSQC experiments, were recorded at 290 K on a 600 MHz NMR spectrometer. The NMR analysis was done using a “blinded” approach whereby the mass or sequence of the peptide was not known by the researcher doing the analysis until after the sequence tag was identified.

Individual spin systems were identified based on the TOCSY and COSY spectra. This process relied on the differences in the amino acid side chains resulting in different patterns in the spectra, and knowledge of the characteristic chemical shifts for particular residues [9]. For example, residues, such as Val, Ile, Leu, Pro, Arg, and Lys, can often be identified based on the cross-peaks and chemical shifts observed in the TOCSY and COSY spectra. By contrast, residues corresponding to an AMX spin system (i.e., Cys, Asp, Phe, His, Asn, Ser, Trp, Tyr) can be ambiguous. However, for the aromatic residues, the NOESY spectra can often assist in assignment of the residue type. Furthermore, serine residues often have the β-protons shifted downfield as compared to the other AMX spin systems. Following the identification of the individual spin systems, sequential connections were determined based on the NOESY spectra, and a sequence tag corresponding to AMX_AMX_ILE_ARG_AMX_AMX_PRO_LYS_GLY was identified.

The sequence tag was used in the “Sequence Stretches Search” in ConoServer, a database that collates the sequences of cone snail derived peptides and currently has sequence information on more than 2200 peptides [10]. This analysis indicated that the only match in the ConoServer database was conopressin G, which has the same mass as the isolated peptide and a sequence corresponding to CFIRNCPKG [11]. Therefore, in this example, the sequence tag represents the entire sequence, but it has ambiguous residues in four positions.

**Figure 2:**
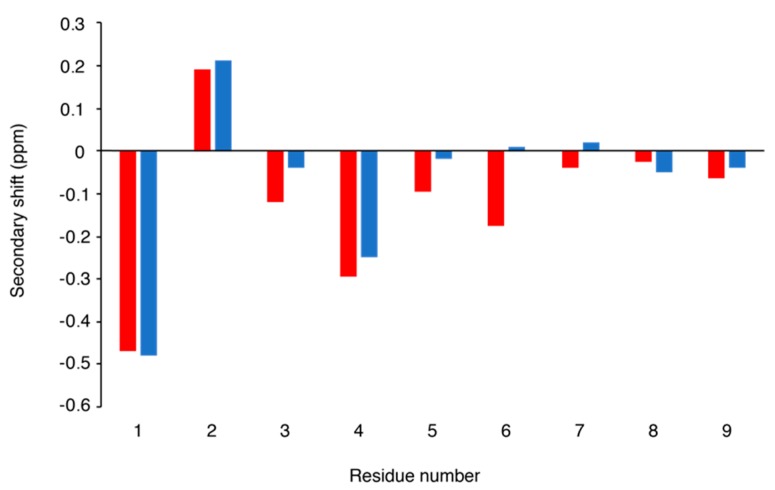
Secondary shift comparison of conopressin T (red) [12] and conopressin G (blue). The secondary shifts were calculated by subtracting random coil shifts [9] from the α-proton shift.

Following elucidation of this sequence, the NMR spectra could be fully assigned. Comparison of the α-proton secondary shifts with conopressin T, a related conopressin that differs in sequence by Y2-F2, Q3-R3, L7-P7, R8-K8, and V9-G9 (Figure 2), indicates that the structure of these peptides is similar [12]. Conopressin T has been shown to be a selective antagonist of the human V_1a_ vasopressin receptor, with partial agonist activity at the oxytocin receptor and no detectable activity at the V_1b_ and V_2_ vasopressin receptors. Additionally, the mutation of Leu7 to a Pro (L7P), which is conserved in all other conopressins that are listed in ConoServer, increased the affinity of conopressin T for the V_1a_ receptor, but had negligible effect on the selectivity across all human receptors [12].

### 2.3. Mass Spectrometry Sequencing of Conopressin G

**Figure 3:**
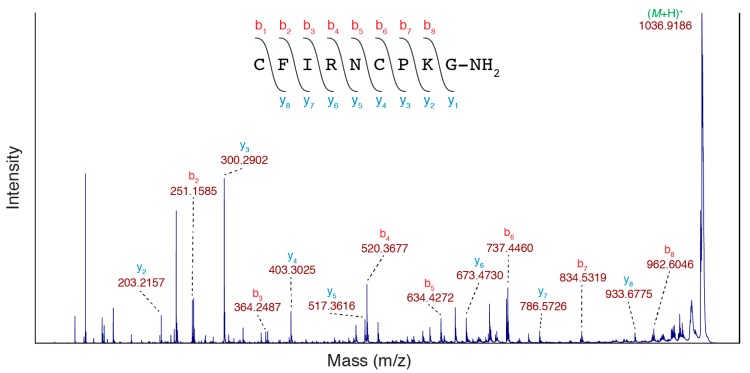
MS/MS sequencing of conopressin G. SCIEX TOF/TOF™ 5800 MALDI MS/MS spectrum of the conopressin G fraction (mass 1033.4459 Da) using 1,5-DAN matrix with the evident y and b ions highlighted.

Although the NMR data was fully consistent with the conopressin G sequence, to validate the NMRseq prediction method, the sequence was also confirmed using MS/MS sequencing. The purified peptide with the mass of 1033.4459 Da was spotted with 10 mg/mL 1,5-diaminonaphthalene (1,5-DAN) and was analysed using MALDI tandem mass spectrometry (MS/MS). The use of 1,5-DAN allows for the reduction of disulfide bonds in the MALDI laser plume and it enhances the in-source decay (ISD) fragmentation of peptides, which can theoretically maintain post-translational modifications [13,14]. 1,5-DAN has been successfully used to sequence venom peptides from a number of species of scorpion and the cone snail *Conus textile* [15,16]. The MS/MS spectrum obtained is shown in Figure 3 and it shows evidence of all y and b ions for the conopressin G sequence with the exception of the y_1_ and b_1_ ions. There is potential ambiguity at residue 3 because the amino acids Ile and Leu have identical masses, however they have unique NMR cross-peak patterns and residue 3 was confirmed as Ile.

### 2.4. Elucidation of the NMR Sequence Tag for G11.1

**Figure 4:**
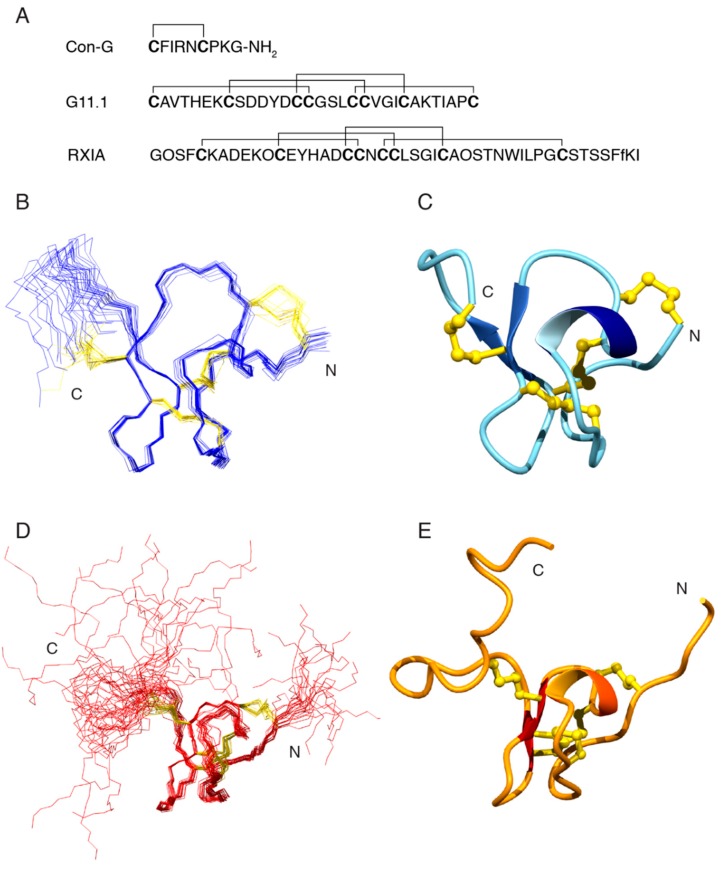
Characterisation of *Conus geographus* venom peptides. (**A**) Amino acid sequences of the two peptides conopressin G (mass 1033.4459 Da) and G11.1 (mass 3202.0503 Da), respectively, and RXIA (theoretical mass 4972.02 Da; O = 4-hydroxyproline, f = d-phenylalanine) with the disulfide bond connectivities illustrated; (**B**) Overlay of the 20 lowest energy three-dimensional NMR structures of G11.1 with the amino acid backbone shown in blue and the disulfide bonds in gold; (**C**) Ribbon representation of the three-dimensional NMR structure of G11.1 showing the presence of the α-helix (navy blue and light blue) and β-sheet (royal blue arrows) secondary structure. Disulfide bonds are shown in gold; (**D**) Overlay of the 20 lowest energy three-dimensional NMR structures of RXIA (PDB 2P4L) with the amino acid backbone shown in red and the disulfide bonds in gold; (**E**) Ribbon representation of the three-dimensional NMR structure of RXIA (PDB 2P4L) showing the presence of the α-helix (orange red and orange) and β-sheet (red arrows) secondary structure. Disulfide bonds are shown in gold. Three-dimensional structure figures generated in MOLMOL [21].

The same approach applied to conopressin G was applied to the peptide corresponding to the mass of 3202.0503 Da, and the following sequence tag was elucidated: VAL_THR_HIS_GLU_LYS_AMX_SER_AMX_AMX_TYR_AMX_AMX. Although the sequence tag consisted of 12 residues/residue types, the VTHEK sequence was unique in ConoServer (accessed on 27 July 2018) and allowed for the identification of the peptide as G11.1 [17]. The complete sequence of G11.1 is shown in Figure 4, and it is consistent with the whole sequence tag obtained. The AMX spin systems were either cysteine or aspartic acid residues.

### 2.5. Three-Dimensional Structure of G11.1

The NMR spectra for G11.1 were fully assigned based on the sequence elucidated in Section 2.3, and the structures were calculated using an automated NOE assignment protocol in CYANA [18]. Dihedral angle restraints were predicted using TALOS-N and hydrogen bond restraints were inferred from the analysis of the temperature coefficients and preliminary structures. Eighteen residues were found to have temperature coefficients more positive than −4.6 ppb/K, indicating that they are involved in hydrogen bonds [19], and restraints for seven hydrogen bonds were included in the structure calculations based on analysis of the preliminary structures.

Structures that were calculated without the disulfide connectivity indicated that Cys 1 is likely bonded to Cys 15, and Cys 14 bonded to Cys 24 based on the distances between the sulfur atoms. The disulfide bond involving Cys 8 was ambiguous and the C-terminal region of the structure was relatively disordered preventing a prediction for the disulfide bond involving Cys 31. To resolve this issue, structures were calculated with the three alternative connectivities involving Cys 8, Cys 19, Cys 20, and Cys 31. The Cys 1-Cys 15 and Cys 14-Cys 24 bonds were also included in the calculations. The CYANA target functions and the disulfide connectivities are shown in Table 1. The CYANA target function was significantly lower for the Cys1-Cys15, Cys14-Cys24, Cys8-Cys20, and Cys19-Cys31, than the two alternative connectivities indicating that this is the most likely disulfide connectivity. Structures were calculated using this disulfide connectivity and the overlay of the ensemble of structures is shown in Figure 4. The main element of secondary structure is a β-hairpin between residues 19 and 25. Four hydrogen bonds were inferred from the temperature coefficients across this β-hairpin.

G11.1 is classified as a member of the I3 superfamily and it contains the XI disulfide framework. RXIA is the only other conotoxin containing this disulfide framework that has been structurally characterized, but it is a member of the I1 superfamily and has a vastly different amino acid sequence with very limited homology with G11.1 (see Figure 4) [20]. RXIA has the same disulfide connectivity as that predicted for G11.1 in the current study. A comparison of G11.1 and RXIA NMR structures is shown in Figure 4.

## 3. Discussion

Venoms from organisms, such as cone snails and spiders, are rich sources of disulfide-rich peptides, many of which are selective and potent modulators of ion channels or receptors [3,6]. Consequently, there is significant interest in analyzing the complex mixtures of peptides that are present in the venoms for a range of applications. These analyses are routinely carried out with mass spectrometry, a technique that has had a significant impact on peptide characterization. However, the mass of a peptide alone does not provide the sequence, and peptides with different sequences can have very similar masses. For instance, the conotoxins SrIA and Qc1.17 differ by only 0.12 of a mass unit, but have no sequence homology (ConoServer [10]). To elucidate sequence information, MS/MS approaches can be used, but for disulfide-rich peptides, this generally requires the reduction of the disulfide bonds and proteolytic digestion. This process can sometimes result in elucidation of the complete sequence, but it is often used to identify sequence tags, which are subsequently searched against transcriptomic or genomic databases to identify the presence of particular peptide sequences. Here, we show that NMR spectroscopy is a complementary approach for the identification of these sequence tags that can subsequently be used for database searching.

Advances in NMR spectroscopy, and in particular, the use of cryoprobes has improved sensitivity [22] to the extent that it is possible to record data on small amounts of native peptide that was directly isolated from venoms. NMR spectroscopy has been used for the analysis of three-dimensional structures of peptides, such as those that were derived from venoms, for decades but generally requires knowledge of the amino acid sequence to interpret the data. However, several types of amino acids have unique characteristics in 2D NMR spectra and for relatively small peptides, with well-resolved spectra it is possible to identify “sequence tags” by connecting specific amino acid spin systems through analysis of NOESY connections.

In the current study, we have exemplified the approach of using NMR to identify sequence tags for two conotoxin peptides, conopressin G and G11.1. The peptides were purified from the complex mixture of peptides present in the *C. geographus* defense-evoked venom and then directly analyzed using 2D NMR experiments. The success of this approach clearly relies on the quality of the NMR spectra. Peptides with multiple conformations and aggregation will likely complicate the process and prevent the assignment of meaningful sequence tags. However, venom peptides are often well structured and soluble in aqueous solution, making this approach highly applicable to this class of compound.

The main advantage of NMRseq is that sequence data is obtained on native sample that can be subsequently used in further analyses, such as calculation of the three-dimensional structure (as the NMR spectra have already been collected) or biological target identification in bioassays. However, it is envisaged that additional applications could include the identification of post-translational modifications, as although transcriptomic and genomic approaches provide a wealth of sequence information, they are limited when it comes to post-translational modifications, which are often present in venom peptides. More comprehensive searches incorporating alternative residues at particular sites could also expand the scope of this approach beyond that which was used with the simple searching used within ConoServer.

Following on from the elucidation of the sequence of G11.1, we determined the three-dimensional structure based on the NMR data, which represents the only structurally characterised member of the I3 superfamily. The structure is similar to that of RXIA, a peptide with the same disulfide framework but a vastly different sequence to G11.1. RXIA targets several sodium channel subtypes [23], but further study is required to determine whether G11.1 also has bioactivity on these channels.

## 4. Conclusions

We have shown here that NMR spectroscopy can be used to identify short sequence tags from native peptide samples that can be subsequently used to elucidate full-length peptide sequences via database searching. We used this NMRseq approach to identify conotoxins from the defensive venom of the cone snail *C. geographus* and determined the three-dimensional structure of a member of the I3 conotoxin superfamily. This approach can be useful for the characterization of disulfide-rich peptides, as they generally have well-defined structures that provide high quality NMR spectra. The primary advantage of NMRseq over other sequencing methods is the provision of native sequence data and the absence of sample degradation in the sequencing process, allowing for subsequent analyses (e.g., bioassays) to be performed with the same sample. NMRseq integrates with, and complements, the proteomic workflow in the venomics pipeline with the distinct advantage of working with intact native sample and maintaining sample integrity.

## 5. Materials and Methods

Commercially available specimens of *Conus geographus* (Cairns Marine, Cairns North, Queensland, Australia) were maintained in an aquarium at a temperature between 24–28 °C. Defense-evoked venom was collected by following the procedure that was outlined in Dutertre et al. [8]. Briefly, the specimen was removed from the tank and pressure applied to the shell until the proboscis extended. A micro-centrifuge tube covered with parafilm was presented to the proboscis until venom was injected through the parafilm. Samples were stored at −30 °C until use.

Crude venom was centrifuged at ~15000× rpm for 10 min in a benchtop centrifuge and the supernatant removed. RP-HPLC was performed and used for purification on a C_18_ semi-preparative column (Phenomenex Jupiter 250 × 10 mm, 10 µm, 300 Å; Phenomenex, Torrance, CA, USA) with a flow rate of 3 mL/min using a linear gradient of 0–60% solvent B (solvent A: 0.05% TFA; solvent B: 90% acetonitrile, 0.045% TFA) over 120 min on an Agilent 1260 series instrument (Agilent, Hanover, Germany). UV absorbance was monitored at 214 nm and 280 nm, and 30 s fractions collected in 2 mL Axygen deep well 96-well plates (Corning, New York City, NY, USA).

Mass spectrometry (MS) was performed using a SCIEX TOF/TOF™ 5800 MALDI mass spectrometer (SCIEX, Framingham, MA, USA). Samples were spotted on 384-well stainless steel target plates using 0.5 μL of sample and 0.5 μL of either α–cyano-4-hydroxycinnamic acid (CHCA; Sigma-Aldrich, St. Louis, MO, USA) matrix at 7.5 mg/mL in 50% ACN/0.1% TFA, or 1,5-diaminonaphthalene (1,5-DAN; Sigma-Aldrich, St. Louis, MO, USA) matrix at 10 mg/mL in 60% ACN/0.04% TFA. Calibration was performed before spectra collection for each sample using Calibration Mix solution 2 (SCIEX, Framingham, MA, USA). Spectra were acquired in reflector positive ion mode from *m/z* 800 to 4500 Da, and averaged over 2000 laser shots. MS/MS spectra were acquired with a collision energy of 2 kV and were averaged over 2500 laser shots.

Lyophilized peptides were resuspended in 90% H_2_O:10%D_2_O. 2D ^1^H-^1^H TOCSY, ^1^H-^1^H NOESY, ^1^H-^1^H DQF-COSY, ^1^H-^15^N HSQC, and ^1^H-^13^C HSQC spectra were acquired at 290 K and 305 K using a 600 MHz AVANCE III NMR spectrometer (Bruker, Karlsruhe, Germany) equipped with a cryogenically cooled probe. All spectra were recorded with an interscan delay of 1 s. NOESY spectra were acquired with mixing times of 200–250 ms, and TOCSY spectra were acquired with isotropic mixing periods of 80 ms. Two-dimensional spectra were collected over 4096 data points in the f2 dimension and 512 increments in the f1 dimension over a spectral width of 12 ppm. Standard Bruker pulse sequences were used with an excitation sculpting scheme for solvent suppression. NMR assignments were made while using established protocols [24], and the secondary shifts derived by subtracting the random coil αH shift from the experimental αH shifts [9]. The two-dimensional NOESY spectra were automatically assigned and an ensemble of structures were calculated using the program CYANA [18]. Torsion-angle restraints from TALOS+ were used in the structure calculations. The final structures were visualized using MOLMOL [21].

## Figures and Tables

**Table 1:** CYANA target function for possible disulfide connectivities for G11.1.

Disulfide Connectivity	CYANA Target Function
Cys1-Cys15, Cys14-Cys24, Cys8-Cys20, Cys19-31	0.076 ^1^
Cys1-Cys15, Cys14-Cys24, Cys8-Cys19, Cys20-31	6.00
Cys1-Cys15, Cys14-Cys24, Cys8-Cys31, Cys19-20	8.47

^1^ Average target function calculated using CYANA [18].
